# DNA Methylation Characteristics of Primary Melanomas with Distinct Biological Behaviour

**DOI:** 10.1371/journal.pone.0096612

**Published:** 2014-05-15

**Authors:** Szilvia Ecsedi, Hector Hernandez-Vargas, Sheila C. Lima, Laura Vizkeleti, Reka Toth, Viktoria Lazar, Viktoria Koroknai, Timea Kiss, Gabriella Emri, Zdenko Herceg, Roza Adany, Margit Balazs

**Affiliations:** 1 Department of Preventive Medicine, Faculty of Public Health, University of Debrecen, Debrecen, Hungary; 2 MTA-DE- Public Health Research Group, University of Debrecen, Debrecen, Hungary; 3 International Agency for Research on Cancer, Section of Mechanisms of Carcinogenesis, Epigenetics Group, Lyon, France; 4 Department of Dermatology, Faculty of Medicine, University of Debrecen, Debrecen, Hungary; Institut national de la santé et de la recherche médicale, France

## Abstract

In melanoma, the presence of promoter related hypermethylation has previously been reported, however, no methylation-based distinction has been drawn among the diverse melanoma subtypes. Here, we investigated DNA methylation changes associated with melanoma progression and links between methylation patterns and other types of somatic alterations, including the most frequent mutations and DNA copy number changes. Our results revealed that the methylome, presenting in early stage samples and associated with the BRAF^V600E^ mutation, gradually decreased in the medium and late stages of the disease. An inverse relationship among the other predefined groups and promoter methylation was also revealed except for histologic subtype, whereas the more aggressive, nodular subtype melanomas exhibited hypermethylation as well. The Breslow thickness, which is a continuous variable, allowed for the most precise insight into how promoter methylation decreases from stage to stage. Integrating our methylation results with a high-throughput copy number alteration dataset, local correlations were detected in the MYB and EYA4 genes. With regard to the effects of DNA hypermethylation on melanoma patients' survival, correcting for clinical cofounders, only the KIT gene was associated with a lower overall survival rate. In this study, we demonstrate the strong influence of promoter localized DNA methylation changes on melanoma initiation and show how hypermethylation decreases in melanomas associated with less favourable clinical outcomes. Furthermore, we establish the methylation pattern as part of an integrated apparatus of somatic DNA alterations.

## Introduction

DNA methylation, along with covalent histone posttranslational modifications, chromatin remodelling and non-coding RNA-mediated gene interference, represents an important mechanism in the integrated apparatus of epigenetic regulation [1], [2]. In addition to playing a role in several physiological processes [3], [4], [5], epigenetic mechanisms have been described as key factors in modifying the accessibility of DNA to transcription factors and, therefore, in altering the gene expression patterns of several cancer types [6], [7], [8]. Given the existence of relatively simple approaches that require even minute amounts of tumour DNA, the best factor described involved in melanoma epigenetics is DNA methylation, a covalent modification of mainly cytosines. The DNA hypermethylation is usually strictly localised to the transcriptionally active gene regions and promoters and directly inhibits gene expression. In the field of malignant melanoma epigenetics, there are substantial amounts of data available regarding gene silencing associated with the localised CpG hypermethylation of specific gene promoters. However, most of the provided data are derived from cell lines or were generated using single-gene approaches [9], [10], [11], [12], [13], [14]. Despite the fact that some groups have attempted to conduct array-based experiments, to date, there are no methylation markers of the diverse melanoma subgroups based on a stratified analysis with sufficient statistical power [1]. Therefore, having chosen a powerful and high-throughput bead array technology, we performed array-based experiments to define the methylation pattern of 1,505 gene promoters. Previous studies have provided irrefutable proof of the reproducibility of this approach [6], [7], [15], [16]. The simultaneous detection of transposonal demethylation and promoter methylation changes should provide valuable information regarding the molecular mechanisms potentially responsible for the aggressive phenotype of malignant melanoma. Recently, it has become widely accepted that Knudson's two-hit hypothesis is often confirmed through a combination of differing types of genomic alterations [17], [18], which prompted us to investigate whether methylation patterns are associated with other types of somatic alterations, such as the most frequent mutations (BRAF and NRAS) and DNA copy number (CN) alterations. Notable previous investigations demonstrated the prognostic relevance of CN aberrations [19], [20], [21], [22]. Therefore, we also highlighted the cis-and trans-acting CN alterations of gene expression in malignant melanoma [23]. Moreover, we and others have demonstrated the association of BRAF and NRAS mutations with CN alterations using BAC arrays, suggesting a central role of BRAF mutations in gene copy number changes [21], [24],[25]. Additionally, a single group reported that the impact of BRAF signalling on gene methylation is widespread [26]. Despite the promising initial results, to our knowledge, no direct, array-based experiments have been performed in an integrative approach in a wide variety of primary melanomas. Therefore, we aimed to obtain better insight into how the DNA methylation changes are associated with distinct somatic alterations and contribute to melanoma progression.

## Materials and Methods

### Melanoma samples

Forty-two primary melanomas were included in Illumina bead assays. The clinicopathological data of the primary melanomas are summarised in Table 1.

**Table 1:** Clinical-pathological parameters of primary melanomas.

Variables	No. of tumours analysed by Illumina bead assay
All patients	42
Histological subtype	
SSM^1^	26
NM^2^	16
Gender	
Female	20
Male	22
Age (years)	
20–50	14
≥50	28
Breslow thickness (mm)^3^	
≤4	26
>4	16
Breslow thickness (mm)^4^	
≤2	15
2–4	11
>4	16
Location of primary tumour	
Extremity	21
Trunk	20
Head	1
Metastasis formation^5^	
Absent	20
Present	22
Patient's survival^6^	
Alive	21
Exitus	21
Ulceration	
Absent	20
Present	22
BRAF^V600E^ mutation	
BRAF^V600E^ mutant	12
BRAF^V600E^ wild type	24

1 Superficial spreading melanoma.

2 Nodular melanoma.

3 Thickness categories are based on the current staging system.

4 Thickness categories are based on the current staging system.

5 Metastasis of the examined primary tumours.

6 Patients with at least a 5-year follow-up period were included.

The tumour tissues were obtained from the Department of Dermatology, University of Debrecen, Hungary. All human studies were conducted in accordance with the principles outlined in the Declaration of Helsinki, were approved by the Regional and Institutional Ethics Committee of the University of Debrecen Medical and Health Science Centre and were conducted according to regulations (Protocol #2836–2008). Written informed consent was obtained from each patient. The tumour diagnoses were made based on formalin-fixed paraffin-embedded tissue sections using haematoxylin and eosin staining. The melanoma tumour staging was determined according to the new melanoma TNM staging system [27].

### Genomic DNA and total RNA extraction

After surgical excision, the fresh tissues were immediately placed in RNA later solution (Applied Biosystems, Foster City, USA), and high-quality total RNA was prepared from the primary melanoma tissues using the RNeasy Mini Kit according to the supplier's protocol (Qiagen, GmbH, Germany). The obtained RNA concentrations were measured using a NanoDrop ND-1000 UV-Vis Spectrophotometer (Wilmington, Delaware, USA). The RNA sample integrity was determined with the Agilent 2100 Bioanalyser using the RNA 6000 Nano Kit (Agilent Technologies, Palo Alto, CA, USA). All RNA samples exhibited a 28S/18S ribosomal RNA ratio greater than 1.5.

The G-spin Genomic DNA Extraction Kit (Intron, Korea) was used to isolate high-molecular-weight DNA from primary melanomas according to the manufacturer's protocol. To determine the quantity of DNA obtained, we used a NanoDrop ND-1000 UV-Vis Spectrophotometer. The DNA integrity was verified via 1.2% agarose gel electrophoresis.

### Bead Assay experiments

The quantitative methylation status of the 1505 CpG sites corresponding to 807 cancer-related gene promoters was determined using the Illumina GoldenGate Methylation Assay (Illumina, San Diego, CA, USA) on bisulphite-treated DNA samples corresponding to 42 primary melanomas. Bisulphite treatment was performed on 500 ng DNA using the EZ DNA Methylation-Gold Kit (Zymo Research). Duplicate samples were included to measure inter-array reproducibility for quality control. The GoldenGate assay consists of two allele-specific oligos (ASO) and two locus-specific oligos (LSO) for each CpG site. Each ASO–LSO oligo pair corresponds to either the methylated or unmethylated state of the CpG site. Each methylation CpG spot is represented by two-color fluorescent signals from the M (methylated) and U (unmethylated) alleles. BeadStudio version 3.2 (Illumina) was used for obtaining the signal values (Avg-Beta) corresponding to the ratio of the fluorescent signal from the methylated allele (Cy5) to the sum of the fluorescents signals of both methylated (Cy5) and unmethylated alleles (Cy3), 0 corresponding to completely unmethylated sites and 1 to completely methylated sites. In agreement with the literature, 83 probes corresponding to the sex chromosomes were excluded to avoid any sex-specific bias [8]. The probes with detection P values exceeding 0.01 in more than 10% of the specimens were removed from the analyses to exclude non-biological differences. As Du et al. performed a systematic comparison between Avg-Beta values and M-values, which is the logit transformation of Avg-Beta, and M-values were proven to be statistically valid for conducting differential methylation analysis [28], M-values were used for class comparisons, whereas the raw Avg-Beta values were applied for correlation analyses (see at “Statistical Analysis”).

### Array CGH for studying copy number alterations

The results data of our previous Tiling Array CGH (HG18 CGH 4×72K WG Tiling v2.0) experiments (Roche NimbleGen core facility, Reykjavik, Iceland) were used which can be found under the following accession number: E-MTAB-947 (Array Express Archive repository).

The GISTIC algorithm was used to identify regions containing a statistically high frequency of copy number aberrations compared to the “background” aberration frequency. This function is most appropriate for cancer samples, as it was designed using a cancer dataset [29]. After the gain/loss aberrations were identified in each sample, a statistic (the G score) was calculated for each aberration. This G score is a measure of the frequency of occurrence of the aberration and the magnitude of the copy number change (log ratio intensity) at each location in the aggregate of all samples in the dataset. Each location is scored separately for gains and losses. The locations in each sample are permuted, simulating data with random aberrations, and this random distribution is compared to the observed statistic to identify scores that are unlikely to occur by chance alone.

Array CGH results were verified using four colour FISH (Abott Molecular Inc., USA). The FISH experiments were performed according to the Suppliers' protocol and visualized by Zeiss Axio Imager Confocal Microscopy.

### Quantitative RT-PCR

The expression status of selected genes (FGFR3, MCAM and IL8) was measured using quantitative real-time PCR with the ABI Prism 7900HT Sequence Detection System (Applied Biosystems, Carlsbad, California, USA). Reverse transcription (RT) was carried out on total RNA (600 ng) using the High Capacity cDNA Archive Kit, according to the protocol of the supplier (Applied Biosystems, Carlsbad, California, USA). Predesigned TaqMan Gene Expression Assays (Applied Biosystems, Carlsbad, California, USA) were used to perform qPCR for the abovementioned 3 genes.

### Statistical analyses

#### The effect of localised methylation on clinical predictors, BRAF^V600E^ mutation and patient survival

We applied random variance t-statistics on all the binary data classes such as Breslow thickness with the cut-off value of 4 mm; metastasis, ulceration and histologic subtype. Being continuous variable, Breslow thickness can be divided into more subgroups: according to the TNM system up to 5 groups can be created, however, due to the limitation of smaller samples, developing 3 groups based on the cut-off values of 2 mm and 4 mm were the most ideal. F-statistics was applied on the trichotomised Breslow groups for each CpG site.

CpG sites were considered differentially methylated when their p values based on univariate t-tests or f-tests were less than 0.01; in addition, given CpG sites were identified differentially methylated between the melanoma subgroups based on a multivariate permutation test providing 90% confidence that the false discovery rate was less than 20%. Volcano plots were applied to illustrate differential methylation patterns among clinical subgroups of melanomas (the clinicopathological characteristics of melanomas are summarized in Table 1). Volcano plots combine p-values of the t-tests for each CpG sites and ratios between the melanoma subgroups. Additionally, the trichotomised Breslow thickness groups were visualized by heatmap and compared by Principal Component Analysis (PCA).

For the aforementioned class comparisons, M-values, the logit transformations of signal intensities were used.

To evaluate the KEGG-based gene networks disturbed by DNA methylation, we applied the Efron-Tibshirani Gene Set Analysis that uses ‘maxmean’ statistics to identify gene sets expressed differentially among predefined classes [30]. The threshold for determining significant gene sets was 0.01 in each approach.

The Cox proportional univariate approach was performed on each gene to test whether the methylation status of a particular gene significantly influences the survival at the p<0.05 level. To control for covariates on survival and to predict the survival risk, the Supervised Principal Components method was used.

As normal tissues were not involved in or studies we used external dataset from the study of Conway et al. involved 27 naevi [15] to check the methylation status of a given gene in control tissues.

Remaining statistics were performed using SPSS 19.0 and GraphPad Prism 6.0 demo version. Venn diagram was made by VENNY, an interactive tool for comparing lists with Venn Diagrams developed by Oliveros, J.C. (2007). The tool is available at: http://bioinfogp.cnb.csic.es/tools/venny/index.html.

#### Relationship between methylation patterns and copy number alterations

We studied how DNA copy number changes and methylation pattern associated within the same genetic loci. For this purpose, the copy number and localised methylation data of the corresponding genomic regions were simultaneously analysed gene-by-gene using CGH Tools, and Pearson's correlation was performed with p<0.01 after correction for multiple testing. Additionally, Fisher's exact test was applied to identify the genome sequences where gene methylation occurs frequently.

## Results

### Methylation patterns of melanoma subgroups

Our experimental design for applying the Illumina Bead Assay included two replicate samples among arrays to measure the inter-array reproducibility. Technical replicates were significantly correlated with each other by Pearson's correlation (Replicate #1: r^2^ = 0.95, p<0.001; Replicate #2: r^2^ = 0.98, p<0.001).

After the initial filtering process, 895 CpG sites were available for further analyses and M-values, logistically transformed Avg-Beta values, were used for statistical approaches.

Our main goal was to investigate the relationship between the distinct biological types of melanomas and the promoter methylation levels. As the multivariate permutation test provides a tight probabilistic control on the proportion of false discoveries, this test was used for class comparison on each predefined subgroup (the clinical subgroups of primary melanomas are detailed in Table 1) according to the following criteria: CpG sites were considered differentially methylated when their p values were less than 0.01 and FDR rates were below 0.2.

**Figure 1:**
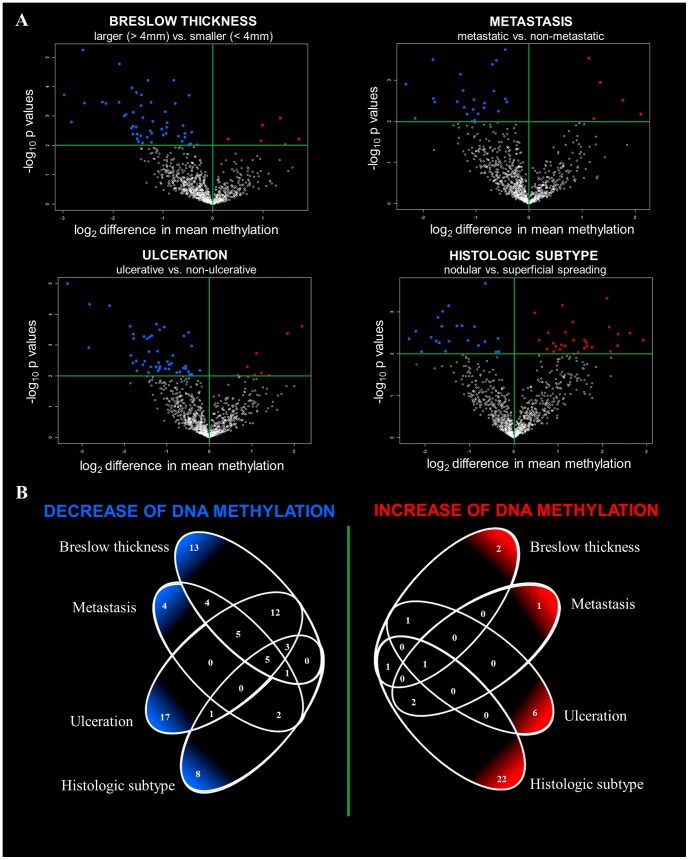
Methylation patterns of primary melanomas associated with known clinical predictors. (**A**) Volcano plots of differentially methylated genes associated with known predictors. Blue dots indicates decreased and red indicates increased methylation as follows: Breslow thickness: 51 hypomethylated probes (43 individual genes) and 5 hypermethylated probes (5 individual genes); metastatic capacity: 23 hypomethylated probes (21 individual genes) and 5 hypermethylated probes (4 individual genes), ulceration: 48 hypomethylated probes (43 individual genes) and 8 hypomethylated probes (8 individual genes), histologic subtype: 28 hypomethylated probes (26 individual genes) 23 hypermethylated probes (20 individual genes) (**B**) A Venn diagrams indicate the overlap of differentially methylated genes (in left: number of hypomethylated genes; in right: number of hypermethylated genes) for each clinical predictor class.

Figure 1A demonstrates that relatively large number of CpG sites was found to be differentially methylated between melanoma subgroups. Interestingly, the majority of these CpGs were characterised by decreased DNA methylation levels in samples with poor prognosis (larger than 4 mm, metastatic, ulcerated and nodular primary melanomas). Histologic subtype exhibited a more disturbed methylation pattern involving high number of differentially methylated genes in both superficial and nodular subtype. As it can be seen in Figure 1A, some of the differentially methylated individual genes were represented by more than one significant CpG sites arguing in favour of the consistency of given alterations. Altogether, 111 differentially methylated genes were identified in the context of aforementioned clinical predictors: 43 individual genes were hypermethylated and 68 genes hypomethylated in melanomas with less favourable clinical outcome. The differentially methylated gene lists specific for the Breslow thickness, ulceration, metastatic capacity and histologic subtype are detailed in Table S1. Venn diagrams (Figure 1B) indicate the common properties among genes with decreased and increased DNA methylation, respectively.

**Figure 2:**
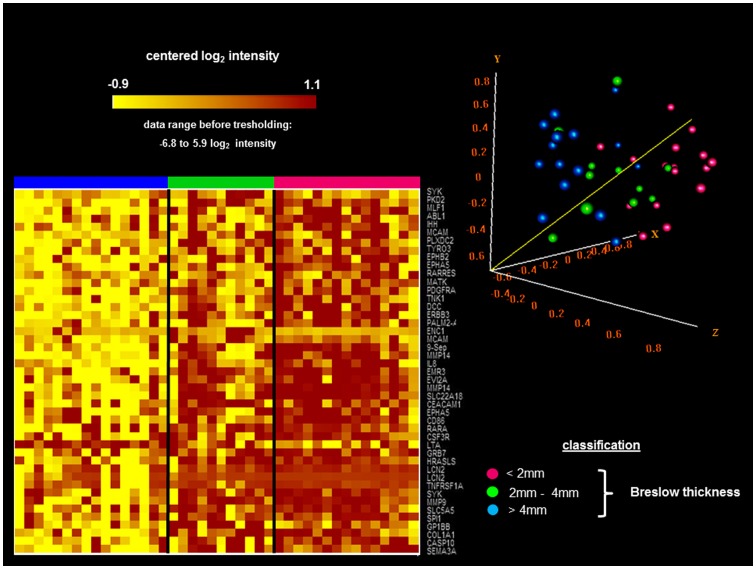
Hypermethylation is an early event in melanomas and decreases with tumour thickness. (**A**) The heatmap demonstrates the hypermethylation patterns (indicated in brown colour) of 45 CpGs, which can be detected in the early stages of melanomas (horizontal purple colour) but decrease from the medium stage (horizontal green colour) to the late stage (horizontal blue colour). (**B**) The principal component analysis for the distinction of the Breslow thickness the sample groups (large: blue dots; medium: green dots; and small melanoma samples: purple dots) based on the 45 differentially methylated CpGs. The analysis revealed that, according to the first three components, which covered the 56% of the total variance, the three groups were significantly different (p<0.05)

Being a continuous variable, Breslow thickness allowed the most precise insight into how methylation pattern changes across melanoma stages. In Figure 2A, the heatmap horizontally shows the primary melanoma samples with distinct Breslow thicknesses. The intensive hypermethylation of 45 CpGs is marked with brown colour in the early stage tumours (Breslow thickness <2 mm), and this hypermethylation decreases during the medium and advanced stages. Low-level methylation values are represented with yellow colour. In other types of cancer, hypermethylation has been shown to be associated with tumour progression. Interestingly, the hypermethylation patterns of 45 CpGs, which are detected in the early stages of melanomas, gradually decrease in the medium stages and almost disappear in late stages of the disease. The Principal Component Analysis (Figure 2B) clearly demonstrated that, according to the pattern of the 45 hypermethylated CpGs, the melanoma groups were significantly separated.

It is important to note that normal tissues were not used in our experiments. However, such datasets can be found in the literature, and we were therefore able to correct for the methylation status of normal naevi specimens (see Materials and Methods). These results thus argue that the hypermethylation of the 45 CpGs occurs early, in melanomas less than 2 mm, and then decreases during melanoma progression.

**Figure 3:**
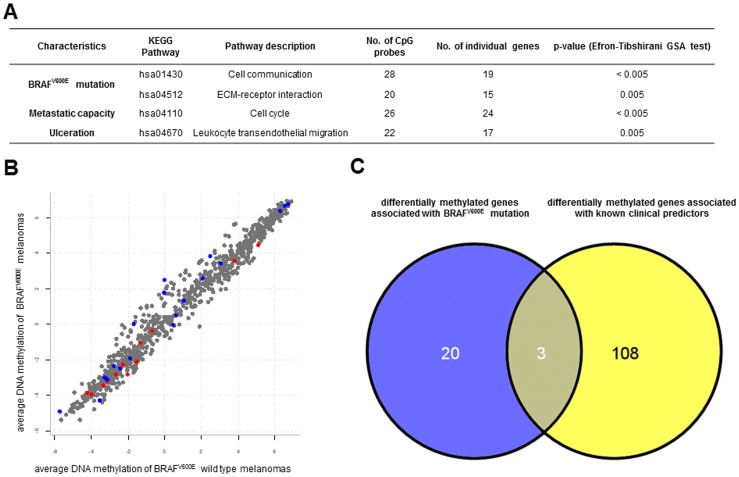
Differentially methylated gene sets between the BRAF^V600E^ mutant and wild-type classes. (**A**) Differentially methylated gene sets between BRAF^V600E^ mutant and wild-type, metastatic and non-metastatic, ulcerated and non-ulcerated classes according to the Kyoto Encyclopaedia of Genes and Genomes. (**B**) Average log-ratios of methylation intensities in BRAF^V600E^ mutant and wild-type melanomas. Red indicates significant genes associated with ECM-receptor interaction and blue depicts significant genes on Cell communication pathway. (Eleven genes overlap between the ECM-receptor interaction and Cell communication.) (**C**) Venn diagram shows lack of overlap between differentially methylated genes associated with BRAF^V600E^ mutation and the known clinical predictors as Breslow thickness, metastatic capacity, ulceration and histologic subtype.

In addition to individual gene signatures, we aimed to determine whether the perturbed KEGG-based gene networks are related to localised methylation patterns. We identified differentially methylated genes belongs to Cell cycle pathways in primary melanomas with metastatic capacity. Genes associated at Leukocyte signalling were also demonstrated to be differentially methylated in ulcerated samples (Figure 3A). Interestingly, Cell communication and ECM-receptor interaction networks were found to be significant at the 0.01 level between BRAF^V600E^ mutant and wild type samples, notwithstanding the fact that, we were unable to find differentially methylated CpGs at the individual gene level (Figure 3A–B). The full list of CpG probes is given in Table S2. There was poor overlap (Figure 3C) between the differentially methylated genes associated with BRAF^V600E^ mutation and clinical subgroups discussed above.

**Figure 4:**
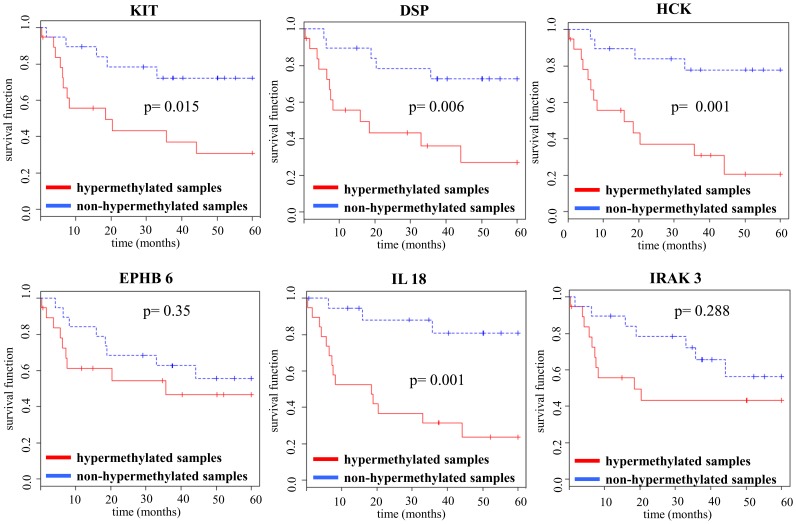
Hypermethylated genes associated with decreased survival rate in melanoma patients. The Kaplan-Meier curves for genes (DSP, EPHB6, HCK, IL18, IRAK3 and KIT) whose hypermethylation was associated with a lower overall survival rate (OS); the Cox proportional univariate approach was performed on each gene to test whether a methylation status of a particular gene significantly influences the survival at the p<0.05 level.

Our analysis of the effects of hypermethylation on patient survival identified an association between six hypermethylated genes (DSP, EPHB6, HCK, IL18, IRAK3 and KIT) with lower OS values. Four of the six genes (DSP, HCK, IL18 and KIT) exhibited significantly different Kaplan-Meier curves (Figure 4). However, when we included patient age, gender and BRAF^V600E^ mutation status in the survival risk prediction model, only the KIT gene remained significant.

### Analysis of the mRNA expression level of the differentially methylated genes identified in melanoma

Three genes among the differentially methylated panel were chosen to measure mRNA expression levels by qPCR (FGFR3, MCAM and IL8) according to the following selection criteria: we exclusively focused on genes that had not been previously referred to as methylated in melanomas; furthermore, FGFR3 was chosen in the context of histologic subtype and MCAM of Breslow thickness, while IL8, being a commonly methylated gene among distinct clinical groups was measured across in all categories (Breslow thickness, histologic subtype, ulceration and metastatic capacity).

**Figure 5:**
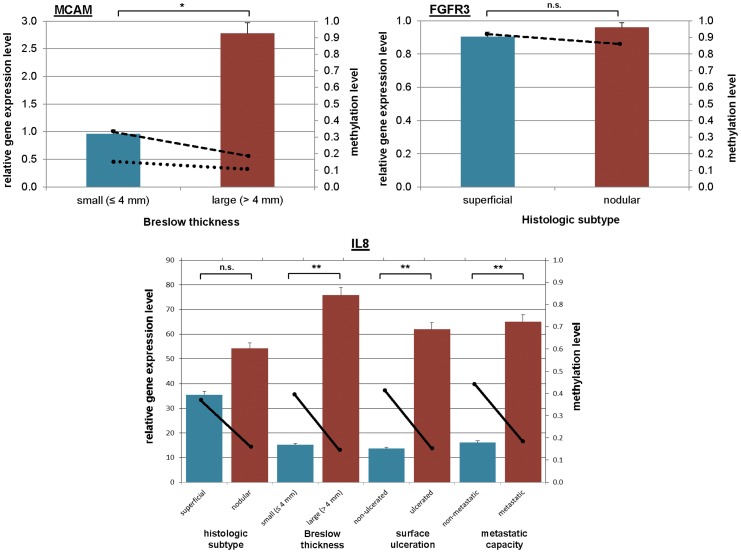
Relationship between gene expression and DNA methylation. The gene expressions of MCAM, FGFR3 and IL8 were measured by qRT-PCR and are presented as bars (fold change in left Y axis), and Avg-Beta methylation values are demonstrated as lines (shown in right Y axis). Methylation data was extracted from Illumina bead assay, with distinct probes represented as different lines. Gene expression differences among the groups were analysed using the Mann-Whitney test, which revealed significant differences for the MCAM and IL8 genes.

Inverse relationships were found between hypermethylation and mRNA expression regarding FGFR3, MCAM and IL8 as well, supporting the notion that the methylation pattern are functionally relevant to gene expression. Significant (p<0.05) MCAM mRNA expression level differences were revealed between smaller (Breslow thickness ≤4 mm) and larger (Breslow thickness >4 mm) melanomas. IL8 expression differed as well between sample distinct categories of Breslow thickness, melanoma surface ulceration and metastatic capacity. The qPCR and corresponding correlation results are summarised in Figure 5.

### Coincidence of localised hypermethylation and copy number alterations

**Figure 6:**
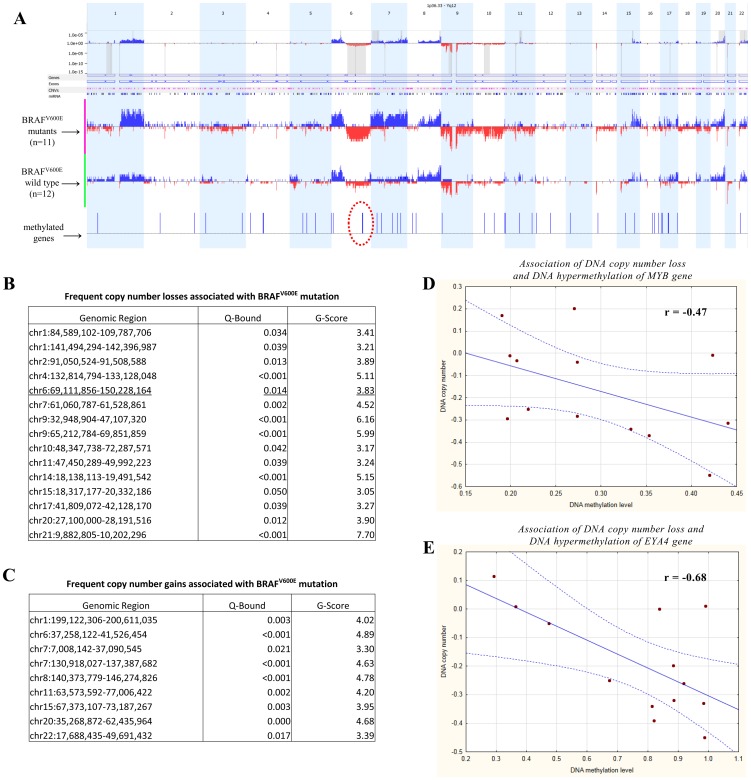
Coincidence of DNA copy number (CN) alterations and hypermethylation. (**A**) The distribution of CN aberrations (red indicates CN losses and blue indicates CN gains on the frequency plot) specific for the BRAF^V600E^ mutant (purple line on the left) and BRAF^V600E^ wild-type (green line on the left) primary melanomas. The methylated genes are shown as blue lines in the lower part of the figure, and the red dotted circle highlights 6q23 as the only region where a coincidence was revealed. The significant CN alterations are highlighted in grey in the upper part of the figure. Frequent CN losses (**B**) and CN gains (**C**) are given based on the G-score of GISTIC algorithm, which is a measure of the frequency of occurrence of the aberration and the magnitude of the CN alteration at each location in the aggregate of all samples in the dataset. The locations of the alterations in each sample are permuted, simulating data with random aberrations, and the significance is represented as Q-Bounds. Grey lines indicate the peak, whereas the grey shaded area is an extended peak based on leave-one-out algorithm to allow for errors in the boundaries in a single sample. (**C**) Correlation plot for CN alterations and DNA methylation regarding the MYB gene and (**D**) the EYA4 gene.

We determined the frequent copy number gains and losses associated with the BRAF^V600E^ mutation (Figure 6A) and Breslow thickness (Table S3) in primary melanomas. As expected, a set of marked copy number alterations was associated with both the BRAF^V600E^ (Figure 6A) mutation and Breslow thickness (Table S3) categories.

In the BRAF^V600E^ mutant samples, significant CN losses (Figure 6B) were found at in the 1p, 1q, 2p, 4q, 6q, 7p, 9p, 9q, 10p, 10q, 11p, 14p, 15p, 17p, 20p and 21p regions, whereas CN gains (Figure 6C) were detected across chromosomes 1q, 6p, 7p, 7q, 8q, 11q, 15q, 20q and 22q.

In the late stages of primary melanomas (Breslow thickness >4mm), significant CN losses were observed more frequently and comprised deletions of 1p, 4q, 7p, 9p, 14p and 21p, whereas CN gains were only observed in the 11q region, as summarised in Table S3. Despite not reaching a significant level, it is worth noting that the CN losses in 19p12 (harbouring the DNA Methyltransferase-1 gene) were exclusively associated with more advanced stages (Breslow thickness >2mm; Figure S1A). However, among the late-stage samples (Breslow thickness >4mm), CN gains were also found with CN losses in some samples. Figure S1B–D represents late-stage melanomas that exhibited CN losses in 19p12.

In addition to the general mapping of the CN-altered genomic regions, we quantitatively assessed the coincidence of CN alteration and methylation patterns gene by gene. Similar to other studies, we established gene level measurements by averaging the methylation states within gene-specific regions. As significantly and positively correlated genes were revealed at the levels of methylation and CN alteration, the correlations cannot possibly represent coordinated allele loss and hypermethylation; nevertheless, these results do not remain significant after the multiple correction procedure. Moreover, direct correlation often involves genome parts that are positively correlated at the level of methylation and CN without detected CN changes or altered methylation. Therefore, we applied an alternative approach based on the frequency of methylated genes harbouring significant CN alterations to test Knudson's two-hit hypothesis. As indicated in Figure 6A, 6q12–6q25.1 comprises a relatively large, significant CN loss and two hypermethylated genes, namely, EYA4 (6q23) and MYB (6q22–q23). When measured quantitatively, a significant inverse correlation was observed between CN loss and DNA hypermethylation (Figure 6 D–E).

**Figure 7:**
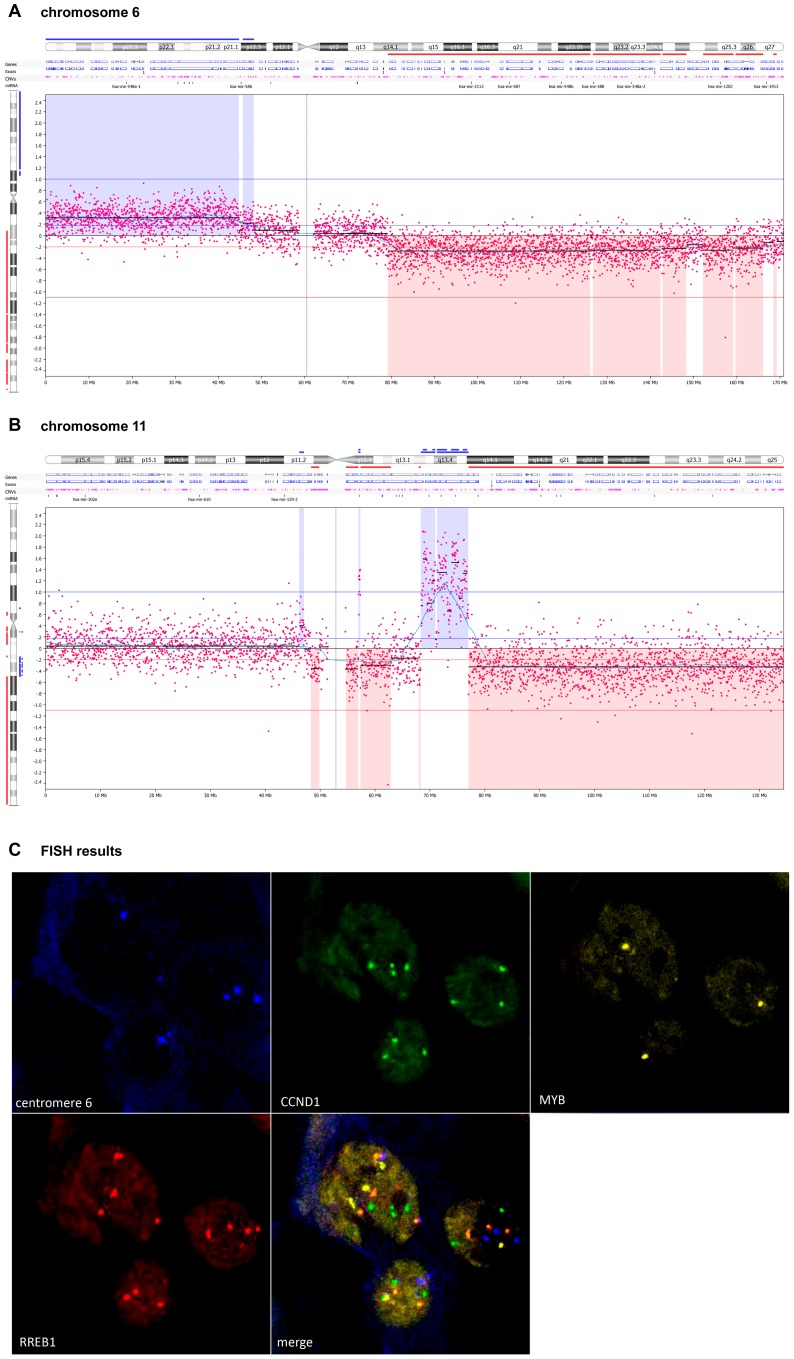
FISH analysis to confirm array CGH results. CN alteration at specific regions of a representative BRAF^V600E^ mutant primary melanoma: (**A**) CN gains were revealed at chromosome 6p while CN losses occurred at chromosome 6q in BRAF^V600E^ samples. (**B**) High level CN gain was seen at the region of 11q13–q14. (**C**) Four colour FISH was performed to verify the CN altered genomic regions: green fluorescence (gain of CCND1 gene on 11q13), yellow fluorescence (loss of MYB gene on 6q23), red fluorescence (gain of RREB1 gene on 6p25), whereas blue fluorescence indicates centromere 6.

Array CGH results were further confirmed by four colour FISH experiments specific for 11q13 (specific for CCND1 gene), 6p25 (specific for RREB1 gene), 6q23 (specific for MYB gene) and centromere 6 on 27 primary melanomas (Figure 7A–C).

## Discussion

Among epigenetic aberrations, DNA methylation itself features a diverse presence [31]. Recently, 5-hydroxymethylcytosine (5-hmC) has been identified as a constituent of mammalian DNA and described as the sixth base of the genome [32]. The loss of 5-hmC has been highlighted as a hallmark of melanoma by a single, remarkable study, whereas interesting clues as to the role of 5-hydroxymethylcytosine are still emerging [33]. In contrast to 5-hmC, the importance of 5-methylcytosine (5-mC) in cancer cells is much more firmly established [1], [34]. Aberrant promoter DNA hypermethylation or localised methylation preferably occurs in CpG dinucleotide-dense regions, resulting in the down-regulation of the corresponding gene [1], [14].

It has recently become apparent that malignant melanomas feature hypermethylation, and currently more than 80 genes – mainly in promoter regions – are hypermethylated at a single-gene level [1], [12], [31]. Taking a global view of the available data, the number of primary tumour samples involved in the studies and the frequency of positive results do not allow determining whether the hypermethylated genes described are appropriate for diagnosis or can be considered candidate therapeutic targets. Moreover, most of the data provided are derived from cell lines and estimated methylation values indirectly consisting of three steps: measuring mRNA or protein expression in cell lines, treating samples with a specific drug that acts against the process of methylation and measuring gene expression again. Nonetheless, powerful arguments have been presented in the literature that support direct experiments being less ambiguous; furthermore, most of the groups conducting direct measurements have applied candidate gene approaches [31], [35].

In addition to the rapid progress that has been made in studying promoter hypermethylation at the single-gene level, only two groups have attempted to conduct array-based experiments to identify the methylation pattern of thousands of gene promoters [15], [36]. Regrettably, one group has focused only on comparing the methylation level of primary invasive melanomas with benign melanocytes and has clearly identified a group of genes in a statistically powerful interpretation that can be used to discriminate naevi from melanomas based on their methylation signature [15]. Another group has examined the short-term cultures of homogeneous stage III specimens [36].

As no data are currently available regarding the methylation markers of diverse melanomas with different clinical behaviours, we performed a systematic comparison of localised methylation patterns among 42 primary melanomas using the Illumina Golden Gate Cancer Panel Bead Assay. We found 111 differentially methylated CpGs altogether among melanoma subgroups and the majority of CpG sites were hypermethylated in melanomas that represent more favourable prognoses including a non-ulcerated tumour surface, superficial spreading histological subtype, non-metastatic subgroup and smaller tumour thickness (Breslow thickness <2 mm). Regarding more advanced-stage specimens, the hypermethylome detected in melanomas that represents better prognoses markedly decreased. The decrease in the methylation levels occurred gradually, as the continuous Breslow thickness variables allowed us to distinguish more than two groups among primary melanomas and to map the progress of demethylation during distinct stages (Breslow thickness <2 mm; Breslow thickness 2–4 mm; Breslow thickness >4 mm). The genes involved in demethylation partially overlap among clinical subgroups: five genes (EMR3, SEPT9, IL8, MMP14 and SLC22A18) were found to be commonly demethylated in large (Breslow thickness >4 mm), nodular subtype, ulcerated and metastatic melanomas. The SEPT9 gene is an ovarian tumour suppressor playing a role in cell cycle control [37]; IL8 gene expression is elevated in metastatic melanomas and can increase the level of MMP2 [38]; SLC22A18 has been reported to be down-regulated due to promoter hypermethylation in gliomas [38], [39]; MMP14 has not been found to play a role in melanoma progression thus far. Among the aforementioned clinical groups, the largest similarity (27 overlapping genes) has been detected between the demethylated genes associated with Breslow thickness and ulceration. The histologic subtype represents the most unique methylation pattern, comprising 30 differentially methylated genes between superficial and nodular melanomas.

Our results contrast those of studies describing the hypermethylation patterns of specific genes as tumour progression-related markers based on single gene approaches. However, Conway et al. supported the claim that a covalent change from cytosine to 5-methylcytosine in the promoter region occurs as an early aberration event in melanomas [15]. Notwithstanding, their results highlighted not only the hypermethylated but also the demethylated genes in heterogeneous melanomas compared to naevi. This group reported a lack of similarity – involving only two genes, namely, RUNX3 and SYK – with the previously published data.

Previously, a group published two independent studies regarding in vitro data that demonstrated how the BRAF^V600E^ mutation causes widespread alterations in DNA methylation [26], [40]. Along with Hou et al., we found hypermethylated CpGs accompanied by the BRAF^V600E^ mutation in primary melanomas. In agreement with these observations, we also found distinct methylation pattern in BRAF^V600E^ mutant primary melanomas involving genes of Cell Communication and ECM-receptor interaction networks. A similar association between the BRAF^V600E^ mutation and DNA methylation was described in colon cancer, as methylated samples convincingly represented a distinct subset encompassing almost all cases of tumours with the BRAF^V600E^ mutation [41]. A remarkable study performed by Roon et al. revealed the *BRAF^V600E^* mutation-specific hypermethylation of CpG regions in colon cancer samples by Differential Methylation Hybridization on high-density oligonucleotide microarrays [42]. Interestingly, the authors identified several cancer-related pathways, including the PI3 kinase and Wnt signalling pathways being differentially methylated between BRAF^V600E^ mutant and wild type samples. Additionally, the group found the silencing of *FOXD3 hypermethylated manner*. Based on these studies, authors suggest that a specific epigenetic pattern can contribute to a favourable context for the acquisition of *BRAF^V600E^* mutations [42]. However, further studies are warranted to further clarify the relationship between the mutation and DNA methylation.

In addition to the common mutations, specific patterns of CN alterations have been reported in melanomas characteristic of unfavourable clinical outcomes [21]. Furthermore, it has become obvious that BRAF^V600E^ mutated melanomas display distinct patterns for CN changes, providing the first line of evidence in support of Knudson's two-hit hypothesis [19], [21]. However, none of the published studies attempted to evaluate the relationship between CN alterations and DNA methylation in melanomas. Our group performed a Tiling Array CGH, and, apart from highlighting common CN losses and amplification in the subgroups of primary melanomas, we demonstrated that 6q12–6q25.1 comprises a remarkable CN loss, harbouring two hypermethylated genes on 6q23, EYA4 and MYB1. This result was measured and verified quantitatively and provides evidence for Knudson's two-hit hypothesis at the level of CN loss and DNA hypermethylation. Notably, MYB1 is an important discriminator between melanomas and naevi, as validated by FISH in 123 melanomas and 110 naevi [43], [44]. The copy number deletion of MYB1 is currently used in the diagnosis of melanoma.

Our Tiling Array CGH experiments showed another important feature: the CN alterations of chromosome 19 were only detected in advanced staged primary melanomas. Notably, the altered genomic regions encompass 19p13.2, which harbours the DNMT1 gene (DNA Methyltransferase-1), which plays a role in the establishment and regulation of tissue-specific patterns of methylated cytosine residues [31]. The DNA CN alterations of DNMT1 in advanced stages primary melanomas raise crucial questions: Is demethylation, contributing to clinical outcomes, only a passive consequence of CN loss? Or do CN alterations – as was demonstrated in the context of epigenetic mechanisms and the BRAF^V600E^ mutation – directly control the DNA methylation changes to influence the gene expression patterns of given molecules? Regardless of the reason for changes in methylation, we obtained better insight into how gene expression levels are regulated by DNA methylation: demethylation was associated with increased mRNA levels, whereas hypermethylation was associated with decreased levels.

In summary, we demonstrated the strong influence of DNA methylation changes on melanoma progression. However, hypermethylation, which has been greatly emphasised in the literature, appears to represent more complexity both in melanoma initiation and progression. Additionally, the inhibition of promoter hypermethylation might represent the most promising therapeutic target for the treatment of melanoma, and several types of DNMT inhibitors are currently being developed [35]. Considering the dual role of DNA methylation, further efforts are needed to investigate the importance of such drugs in melanoma treatment.

## Supporting Information

Figure S1 **Copy number alteration of chromosome 19 in Breslow thickness > 4 mm melanomas.** (**A**) The Tiling Array CGH revealed characteristic CN differences among the three sample groups (pink line on the left: Breslow thickness <2 mm; yellow line: Breslow thickness 2–4 mm; red line: Breslow thickness >4 mm) regarding the CN alterations of chromosome 19. The CN-altered regions involve 19p13.2 harbouring the Methyltransferase-1 gene (DNMT1). Panels (**B–E**) depict representative figures of CN losses revealed exclusively in medium- or advanced-stage (according to Breslow thickness) primary melanomas.
(PDF)

Table S1 **Differentially methylated gene lists specific for the Breslow thickness, ulceration, metastatic capacity and histologic subtype in primary melanomas.**
(XLSX)

Table S2 **Differentially methylated genes of Cell communication and ECM-receptor interaction networks in BRAF^V600E^ mutant primary melanoma samples.**
(XLSX)

Table S3 **Copy number alterations associated with Breslow thickness in primary melanomas.**
(XLSX)
