# Supplementary material for: DNA Methylation Characteristics of Primary Melanomas with Distinct Biological Behaviour
Source: PLoS One. 2014 May 15;9(5):e96612. doi: 10.1371/journal.pone.0096612 (PMC4022506; doi:10.1371/journal.pone.0096612)
Supplement: Figure S1 — Copy number alteration of chromosome 19 in Breslow thickness > 4 mm melanomas. (A) The Tiling Array CGH revealed characteristic CN differences among the three sample groups (pink line on the left: Breslow thickness <2 mm; yellow line: Breslow thickness 2–4 mm; red line: Breslow thickness >4 mm) regarding the CN alterations of chromosome 19. The CN-altered regions involve 19p13.2 harbouring the Methyltransferase-1 gene (DNMT1). Panels (B–E) depict representative figures of CN losses revealed exclusively in medium- or advanced-stage (according to Breslow thickness) primary melanomas. (PDF) [file pone.0096612.s001.pdf]

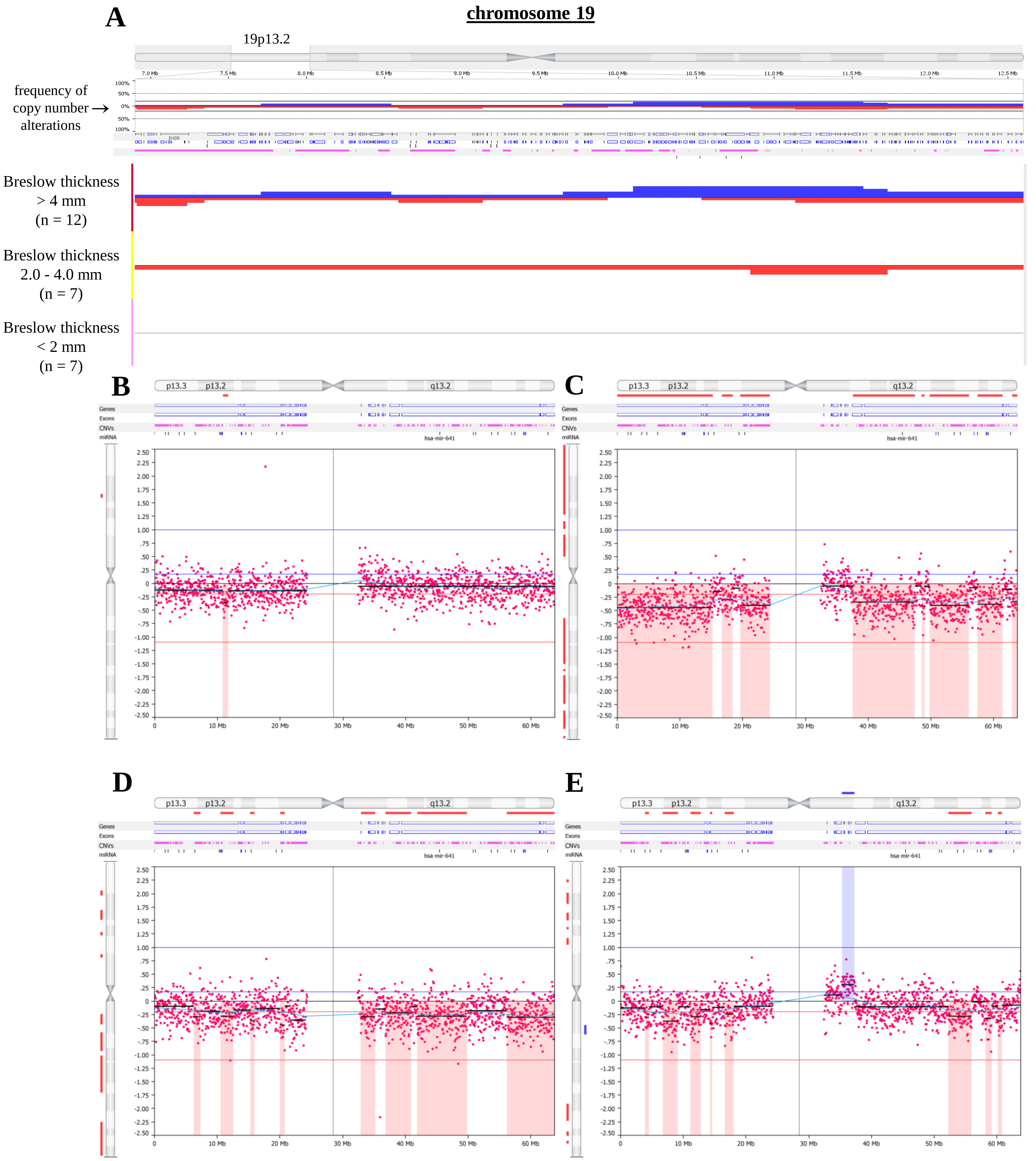

A
chromosome 19
19p13.2
frequency of copy number alterations
Breslow thickness > 4 mm (n = 12)
Breslow thickness 2.0 - 4.0 mm (n = 7)
Breslow thickness < 2 mm (n = 7)
B
C
D
E
p13.3
p13.2
q13.2
Genes
Exons
CNVs
miRNA
hsa-mir-641
